# Quantifying uncertainty in aggregated climate change risk assessments

**DOI:** 10.1038/s41467-021-27491-2

**Published:** 2021-12-08

**Authors:** Luke J. Harrington, Carl-Friedrich Schleussner, Friederike E. L. Otto

**Affiliations:** 1 New Zealand Climate Change Research Institute, Victoria University of Wellington, Wellington, 6012 New Zealand; 2 Climate Analytics, 10969 Berlin, Germany; 3 IRI THESys, Humboldt University, Berlin, Germany; 4 Grantham Institute, Imperial College London, London, UK

**Keywords:** Climate change, Climate-change impacts, Projection and prediction

## Abstract

High-level assessments of climate change impacts aggregate multiple perils into a common framework. This requires incorporating multiple dimensions of uncertainty. Here we propose a methodology to transparently assess these uncertainties within the ‘Reasons for Concern’ framework, using extreme heat as a case study. We quantitatively discriminate multiple dimensions of uncertainty, including future vulnerability and exposure to changing climate hazards. High risks from extreme heat materialise after 1.5–2 °C and very high risks between 2–3.5 °C of warming. Risks emerge earlier if global assessments were based on national risk thresholds, underscoring the need for stringent mitigation to limit future extreme heat risks.

## Abstract (web summary)

The reasons for concern framework are an effective visualisation of climate change related risks. Here, the authors propose a new framework by which different levels of uncertainty can be included into this aggregated assessment in order to ensure a transparent communication of risks.

## Introduction

Extreme weather events are among the most pertinent perils of anthropogenic climate change^1^. There already exists robust evidence that the impacts from changes in the intensity and likelihood of extreme weather events are widespread^2–4^, with extreme heat particularly affecting large fractions of the global population^5–8^. These changes are already visible over the historical period, as robust increases in extreme weather indices over land can already be observed at warming of only half a degree^9^. Recent research on compound^10^ and concurrent^11^ extreme events, together with the fact that relatively moderate extremes can lead to large damages^12,13^, implies that fundamental limits to adaptation to extreme weather events may be reached for parts of the world at lower warming levels than previously thought^14^.

**Fig. 1:**
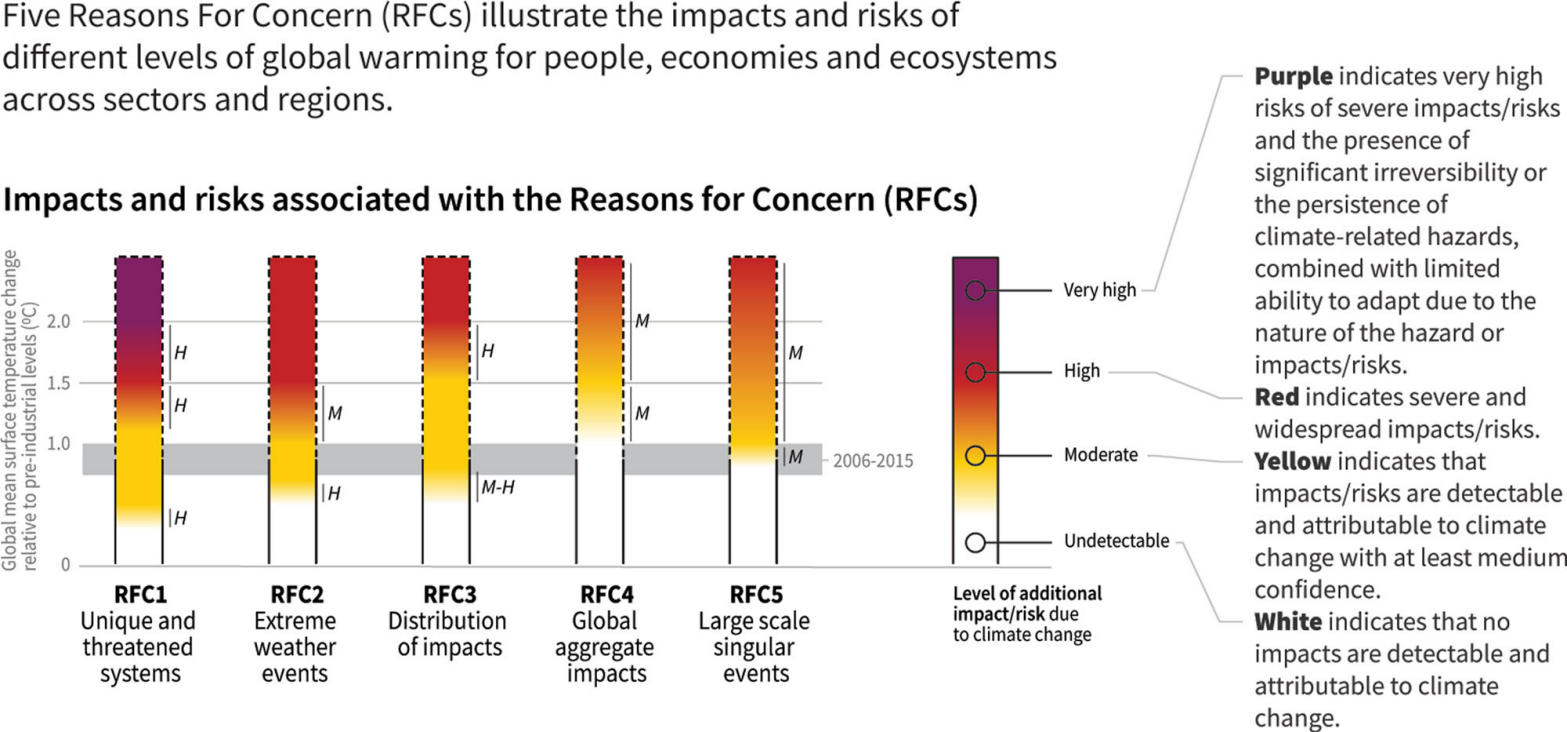
The assessment of impacts and risks of climate change in IPCC Reasons for Concern framework. Reproduction from Fig. SPM2 of the IPCC Special Report on the Global Warming of 1.5 °C (SR1.5)^1^.

Changing likelihoods and intensities of extreme weather events and their impacts are one of five “Reasons for Concern” (RFCs) developed in the context of reports by the Intergovernmental Panel on Climate Change (IPCC), to allow for an aggregated representation of changing climate impacts with rising global temperatures^15,16^. First introduced in the third Assessment Report of the IPCC^16^, subsequent reports including the Special Reports on Global Warming of 1.5 °C^1^ (SR1.5), and Oceans and Cryosphere in a Changing Climate^17^, included important updates to the context (see Fig. 1 for an overview of the RFC framework). The RFC framework communicates scientific understanding about risks relating to varying levels of climate change, and in doing so, represents a critical communication tool for the scientific community^18,19^. As with any approach to aggregation and simplification, a range of challenges and shortcomings of the concept have been discussed. Impacts of anthropogenic climate change are strongly modulated by the vulnerability and exposure^20^ of a community, as well as how emerging climate hazards themselves differ from region to region^21^. Recent reviews of the concept by O’Neill and et al.^18^ and Zommers et al.^19^ have further examined the important role of expert judgement, and the need for improvements on how expert judgement is applied in a systematic fashion. These studies identified a number of research needs to improve the literature base the RFCs depend upon, and thus the RFCs themselves. Three aspects were highlighted: the scientific basis to assess the transitions between different risk levels, the integration of vulnerability and adaptation, and the need for transparency associated with uncertainty in expert judgement.

This perspective addresses these aspects by proposing a transparent methodology to quantitatively identify crossing of thresholds from one risk level to the next while explicitly taking uncertainties from spatial heterogeneity and expert judgements into account.

## Problems with the current framework

In previous assessments, the categories to interpret different risk levels in the RFCs are colour coded^18^: white represents negligible risks; yellow denotes moderate risks, defined as detectable and attributable with at least medium confidence; red denotes severe and widespread impacts; while purple is associated with severe impacts which are either irreversible or persistent, combined with a limited ability to adapt to these impacts.

Two aspects of this framework promote ambiguity when defining the transition between different risk categories. First, the sequential introduction of exposure and vulnerability components as being relevant only for the high and very high risk categories, respectively, complicates the justification to transition between any two risk levels. For example, the number of vulnerable people exposed to the impacts of extreme weather will not instantaneously change from zero to many, when the risk category changes from moderate to high or very high—rather, the numbers will progressively increase as global temperatures continue to rise. Second, the aggregated nature of the different risk categories (most commonly on the global level) do not allow for consideration of different risk thresholds being crossed faster in some regions of the world than others. A simple global risk transition might give the impression that substantial impacts associated with extreme weather suddenly emerge across a large (or “widespread”) proportion of the global population simultaneously, which is not the case. Similarly, high risks at the regional level might occur at lower levels of warming, well before an equivalent transition is experienced globally.

Regional disparities in risk emergence are not only the result of different emergence of physical climate hazards but also need to account for very different vulnerabilities and exposure that contribute to the climate risk. Any efforts to evaluate risk across a breadth of vulnerability profiles, whether between countries or within them, requires particularly careful consideration. While these differential distributions of impacts are explicitly addressed in one of the categories of the RFC framework, RFC3, the question of a meaningful aggregation of very different regional risks remains an important one for all categories.

## A consistent method to quantify the local risks of extreme heat

To address these shortcomings, we present a method that combines spatially resolved climate hazard, population (exposure) and vulnerability indicators to derive local risk categories, before considering how to assign the globally aggregate risk associated with that RFC as a subsequent step. The key focus here is one of the fastest emerging hazards, extreme heat; the number of people exposed to extremely hot days; and the quality of governance and institutions of a country, as a measure of both vulnerability and the capacity for adaptation. Though knowledge about limits to adaptation is still incomplete, governance has been identified as a crucial measure for states to deal with adverse effects of extreme weather, both in the direct aftermath of an extreme event happening as well as long term^22,23^. Clearly, this indicator does not fully capture the multifaceted, multilevel nature of adaptation including not only the state but also the community and household level. But for the aggregated analysis on country and global level presented here, it represents a meaningful proxy for vulnerability and adaptive capacity bearing its limitations in mind. And indeed, it is those states with a low governance index (GI) where extreme weather events are regularly causing widespread damages today^24^ and where new challenges emerging from a changing climate are rarely anticipated^25^.

Characterising the risks associated with extreme weather events would ideally require a multivariate assessment of multiple classes of severe weather. However, to best characterise the geographic signal of hazard changes in a warming climate, while minimising uncertainty associated with the magnitude of future signals, we focus only on emergent increases in extreme daily temperatures. Since changes to extreme heat are emerging faster than other types of weather-related hazards by orders of magnitude^26^, they subsequently dominate the aggregate pattern of emergent hazards that could be considered under the labelling of RFC2 (“extreme weather events”). Discussing the implications of our approach for all RFCs in related key risks goes beyond the scope of this contribution. We note, however, that our proposed framework is also amenable to the introduction of other hazards, and uncertainty relating to the choice of metric is discussed further below.

**Fig. 2:**
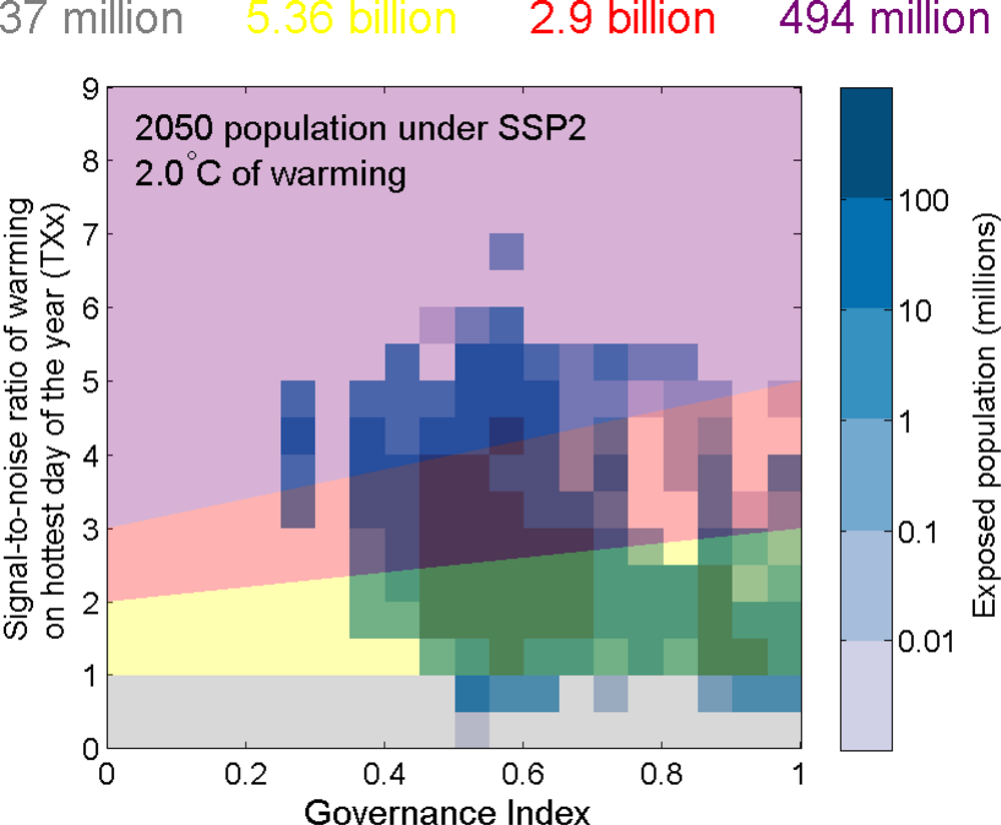
Identifying the local exceedance of heat risk thresholds associated with the “Reasons for Concern” framework. Heat maps here show the range of country-level governance index (*x* axis) and the signal-to-noise ratio of changes in the hottest day of the year (TXx, *y* axis) experienced by different fractions of the global population under one future scenario of warming and socioeconomic change. Population and governance index data are presented for the year 2050 under a middle-of-the-road socioeconomic scenario (SSP2) while the multimodel median exposure to extreme heat (*y* axis) is shown in response to a global temperature rise of +2 °C above early industrial levels. Coloured numbers above the panel denote the total number of people within each of the four risk designations, which are represented by shaded regions in the background of the main figure: purple represents “very high” risks; red represents “high” risks; yellow denotes “moderate” risks and grey denotes “low” risks.

Figure 2 presents an illustration of this method to quantify local population exposure to the risks associated with extreme heat. For each person in the world under a future scenario, the strength of the governance structures in the country in which they live are compared against the magnitude of projected changes in extreme heat, with results for all members of the global population then aggregated. Signal-to-noise (hereafter S/N) ratios in annual maximum daily maximum temperatures (TXx) are presented on the *y* axis, with corresponding GI values on the *x* axis characterising country-level governance as a score between 0 (poor) and 1 (excellent; see ref. ^22^ and “Methods” for further details).

The blue shaded contours in Fig. 2 show the population exposed to the different risk categories under a “middle-of-the-road” (SSP2) population scenario in 2050, and in response to global-mean temperatures reaching 2 °C above early industrial levels (see “Methods”). For simplicity, we hereafter focus on alternative socioeconomic outcomes by the year 2050 only, but explore alternative outcomes for 2090 in Supplementary Information.

The shaded contours in the background of Fig. 2 illustrate how risk is defined to factor in both differences in the slope of changes in extreme heat, as well as regional differences in vulnerability. In line with O’Neill et al.^18^, we have identified all hazard changes smaller than +1σ as non-detectable (grey). For the transition between moderate and high risk, we calibrated the threshold so that it is reached when a country with very poor (excellent) governance experiences a + 2σ (+3σ) shift in TXx, using linear gradients to interpolate across the GI space. Similarly, for the threshold between high and very high risks, we assumed a + 3σ increase in TXx for the lowest GI thresholds, and gradients such that TXx > +5σ represents a crossover into the “very high risk” category for a country with excellent governance (GI of 1) in 2050. There are equally valid arguments to have a negligible gradient for GI scores below 0.5, and stronger increases thereafter. However, given the main aim is to introduce a quantitative methodology rather than presenting a perfect framework, we chose the simpler option. The hazard risk thresholds represent different classes of extreme event emergence linked to the “severe” and “persistence” criteria for the RFC risk level transitions. A + 3σ increase in TXx is roughly equivalent to about a one in hundred to thousand year extreme event, a highly unusual extreme, whereas a + 5σ increase equates to quasi-unprecedented extremes being the new normal and thus the persistence of a new climate regime^27,28^.

Under this scenario, which we also use as a baseline for subsequent analysis, nearly 500 million people are exposed to “very high” risks associated with local changes in extreme heat-related hazards, while 2.9 billion and 5.4 billion people are exposed to “high” and “moderate” risks, respectively.

**Fig. 3:**
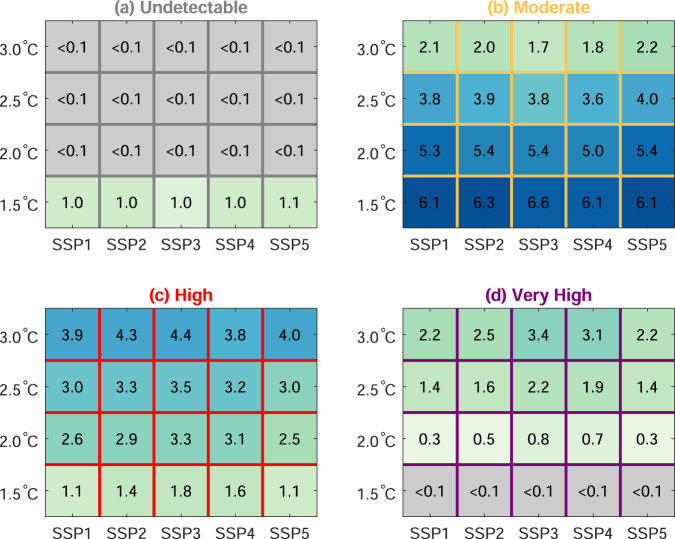
Socioeconomic and emissions scenario uncertainty in local heat risks. Each panel shows the number of people locally exposed to each of the four risk categories designated in Fig. 2, using population data from five socioeconomic scenarios (SSPs) in the year 2050, and calculating exposure levels for a range of specified global warming thresholds. The numbers in the boxes denote billions of people associated with that risk category; the corresponding shading denotes the fraction of the global population associated with that risk category for that particular SSP scenario.

Figure 3 further expands these results, showing the global population exposed to each of the four risk categories by 2050 under five scenarios of socioeconomic development (shared socioeconomic pathways, SSPs), and associated with different prescribed thresholds of global temperature rise. The numbers in Fig. 3 denote billions of people exposed to each of the risk categories, while the background colours illustrate the fraction of the global population in each category. Results show more than two billion people are exposed to “very high” risks associated with extreme heat after 2.5 °C of warming, irrespective of the SSP scenario considered. However, the number of people exposed to the “very high” risk category is estimated to be 50% higher under the SSP3 scenario when compared with corresponding exposure levels under the SSP1 scenario: this reflects the former being described as a “rocky road” scenario typified by regional rivalries and high population growth, while the latter is characterised by lower levels of population growth and more sustainable development pathways^22,29^.

It is noted that data from the socioeconomic scenarios in Fig. 3 remain fixed on values for the year 2050, while the warming thresholds and associated changes in climate hazards are extracted from different time slices of a high emissions scenario throughout the 21st century (following ref. ^30^). The primary value of this exercise is to systematically explore variability in the people exposed to risk categories under a wide range of scenario uncertainty^31^ rather than explicitly considering the viability of these different combinations in the future, which has been done elsewhere^32^.

## Dimensions of uncertainty in aggregated risks

**Fig. 4:**
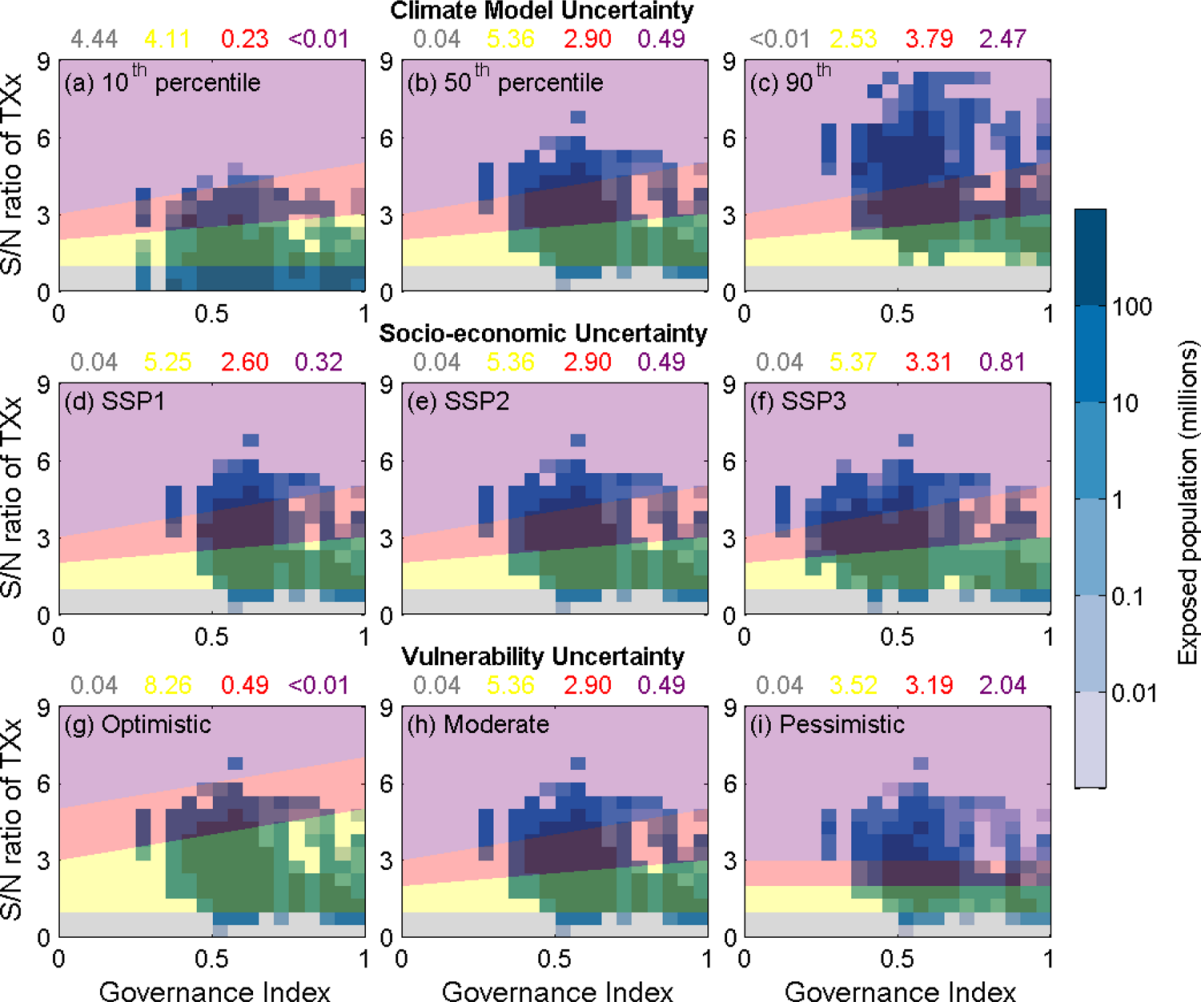
Examining the individual dimensions of uncertainty affecting local risk estimates. As with Fig. 2, the background colours represent the four risk categories (grey is negligible; yellow is moderate; red is high; purple is very high) associated with different thresholds of extreme heat hazards, and governance index thresholds. Panels in the middle column (**b**, **e**, **h**) represent the same scenario as for Fig. 2: the median CMIP5 estimate of the number of people exposed to each of the four risk categories in the year 2050 under SSP2, when global temperatures are 2 °C above early industrial levels. **a** and **c** explore variations in these results due to climate model uncertainty; **d**, **f** explore variations due to alternative SSP scenarios; and **g** and **i** consider differences arising from uncertainty in the vulnerability threshold (as designated with variations of local risk thresholds in the background). The four coloured numbers above each panel denote billions of people within each of the four risk categories.

Threshold-based aggregation is subject to expert and value judgements and we have introduced a set of well-justified but nevertheless subjective criteria to define risk level transitions. In order to allow for transparency on the implications of those choices, a sensitivity analysis is in order to also provide estimates for the overall uncertainty. Our approach, explicitly resolving proxies for the climate hazard (TXx), exposure (population) and vulnerability component (governance), offers an opportunity to disaggregate the relative magnitude of these different dimensions of uncertainty. To do so, we explore in Fig. 4 the dimensions of uncertainty associated with the example presented in Fig. 2, which we hereafter refer to as the “default” profile of risk. The middle column of panels in Fig. 4 present a repeated sequence of the same set of assumptions as in Fig. 2: S/N ratios of TXx using the Coupled Model Intercomparison Project Phase 5 (CMIP5) multimodel median response to 2 °C of warming, under 2050 and a “middle-of-the-road” SSP2 scenario.

We select two alternatives to these default scenarios, termed the “optimistic” and “pessimistic” variants, for each of the different dimensions of uncertainty: (1) that which relates to the spread in CMIP5 model outcomes; (2) uncertainty related to future socioeconomic development (using the SSPs); and (3) “vulnerability” uncertainty, which we propose relates to how local risk thresholds vary in response to both the GI and severity of the climate hazard found in a given location.

All of these uncertainty sources yield variations in the number of people associated with each of the four risk categories, albeit to differing extents and for different reasons. For example, when considering the multimodel 10th (Fig. 4a) and 90th (Fig. 4c) percentile of CMIP5 TXx changes at each grid cell, the number of people locally exposed to the “very high” risk category changes significantly, from an estimated half a billion under the default profile of risk to as few as 1.3 million (models with small changes in extremes) or as many as 2.47 billion people (models with large changes in extremes), signifying a fivefold increase from the default profile in the latter case. These differences in the “high” and “very high” risk category counts between the median and pessimistic outcomes related to model uncertainty are comparable to those found when 3 °C of global warming is experienced by 2050, instead of only 2 °C (Fig. 3).

When comparing across different SSP scenarios (middle row of Fig. 4), the change in population counts associated with each risk category are less prominent. While the bulk of the global population live in countries with poorer levels of governance under SSP3, relative to SSP1, the numbers of people falling within the “moderate” or “high” risk thresholds nevertheless remain similar to the default profile of risk under SSP2. However, these differences do become more pronounced if the same levels of warming are assumed to be reached only in 2090 instead of 2050, as the total global population diverges substantially across the three scenarios later in the 21st century, resulting in much greater changes in subsequent exposure to the different risk categories (Supplementary Fig. 2).

In addition, these SSP-driven differences would become even more pronounced if the considerable uncertainty underpinning the assumptions used to quantify “vulnerability” are taken into account. In fact, when other ways to quantify local risk as a function of GI and TXx are assessed in isolation (bottom row of Fig. 4), the differences in risk exposure between the optimistic and pessimistic vulnerability assumptions are comparable to those associated with climate model or emissions-related scenario uncertainty.

## From local to global risk aggregation

Another layer of expert judgements needs to be introduced to aggregate from local risk assessments to a global level. Here, we choose to define transitions in aggregate risks as a function of the number of people within each of the risk categories. To reveal how these aggregation thresholds represent a further source of uncertainty in quantifying a final risk category, we again consider a range of three equally plausible options. For the “risk-averse”, “risk-neutral” and “risk-tolerant” variants, we arbitrarily select the number of people locally exhibiting the “very high” risk criteria to respectively exceed 0.75, 1.5 or 3 billion people, for the overall risk to be classed at this highest level. Failing this, the aggregate risk will instead be classed as “high” if the number of people exceeding the high or very high risk threshold, exceeds the same thresholds. Similarly, if these criteria are not satisfied, then the aggregate risk will be classed as “moderate” if the number of people with local risks classed as moderate or worse, exceed the same thresholds.

**Fig. 5:**
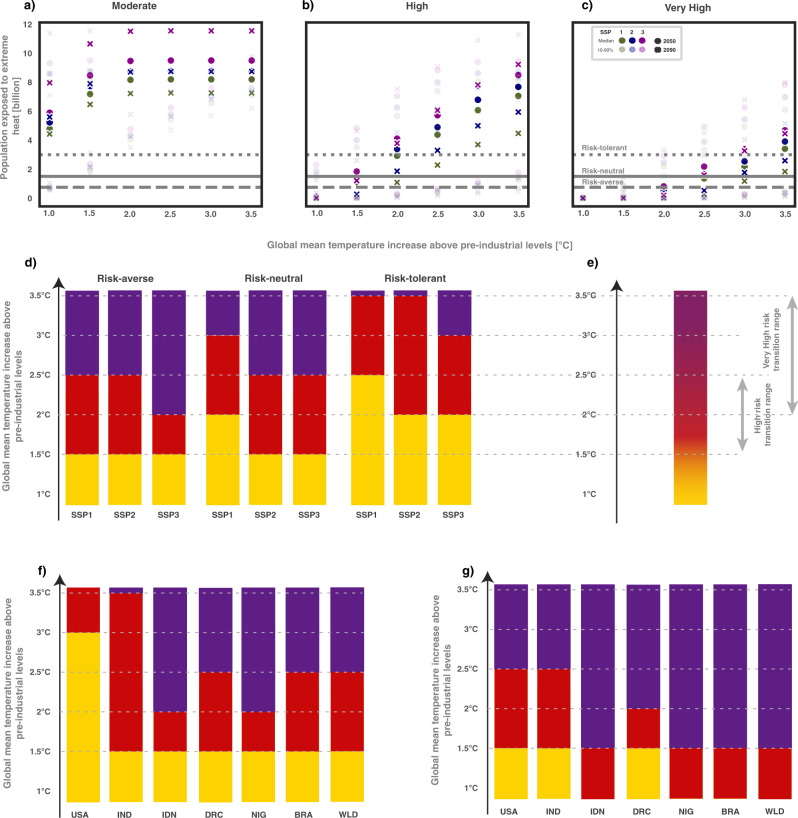
Tracing local risks to develop an aggregate RFC summary. **a**–**c** The number of people experiencing local risks equal to, or worse than, “moderate,” “high” or “very high” categories, at different thresholds of global temperature rise, and considering all dimensions of uncertainty explored in Fig. 4 and Figs. S1–S4. Darker markers show the outcomes for the median CMIP5 results, and using the moderate vulnerability category; the lighter colours show the full range of outcomes. The aggregate risks assignments in **d** are chosen using the darker circles in **a**–**c**, by selecting the highest risk category which is experienced by at least 0.75 (“risk-averse”), 1.5 (“risk-neutral”) or 3 billion (“risk-tolerant”) people for each GMT-SSP combination. **e** shows how the aggregate risk summary (or “burning ember” diagram) is calculated, with uncertainty ranges drawn from the range of plausible outcomes presented in **d**. Regional perspectives downscaling of global risks are presented in **f**. Thresholds for population exposed have been rescaled to the national level relative to the SSP2 2050 population (globally 9.2 billion). Country-level exposure thresholds are set to 16% of the country’s SSP2 2050 population. Country-level risk is shown here for “risk-neutral” thresholds under SSP2 and with a “moderate” vulnerability assumption. **g** A sensitivity assessment of risks starting from a regional perspective for the USA assuming regional “high risks” to be reached at 1.5 °C. The configuration where this is the case is based on a pessimistic vulnerability assumption and aggregation threshold set at 5.5% of population, and risks for other countries and the global level in **g** are assessed using the same methodology.

When exploring the full range of plausible future risks associated with extreme heat (Fig. 5a–c), we find a breadth of outcomes that are consistently worsening with additional warming. Figure 5d presents results for the three different aggregation thresholds, after necessarily reducing uncertainty in the relationship between GI and the emergence of local risks to a “best-guess” estimate. A transition from moderate to high risks occurs in all but the “risk-tolerant” case between 1.5 and 2 °C, similar to the expert assessment presented for RFC2 in the IPCCs SR1.5 report1. Furthermore, our approach also allows for a categorisation of a transition to the “very high” risk category that, accounting for the SSP and threshold uncertainty dimension, can occur after as little as 2 °C, or as much as 3.5 °C of warming.

Our approach allows for a transparent quantification of risks, while at the same time provides for traceability of transition ranges between risk levels to different dimensions of uncertainty: in this case, risk thresholds and socioeconomic scenario development representing changes in dimensions linked to exposure (population) and vulnerability (GI). This allows to not only provide a best estimate for risk transition but also to inform the risk transition ranges (Fig. 5e). When based on a transparent methodology like in the case presented here, the transition ranges linked to RFCs can be efficiently utilised to integrate information about socioeconomic dependencies and other sources of uncertainties of risk transitions. Our approach also allows to illustrate RFCs following a precautionary approach. If, e.g., the upper 90th quantile of climate model uncertainty is considered, “high” risks would be reached already today at 1 °C of warming, with the transition to “very high” risks above 1.5 °C (compare Fig. 5b, c).

Regional or national risk assessments can be instructive when interpreting these global thresholds. Figure 5f provides an illustration of such risks for different countries downscaling the global population thresholds set for the global assessment provided in Fig. 5e. It illustrates how high and very high risks for vulnerable tropical countries emerge at lower levels of warming than the global average, while developed countries spread over temperate and subtropical climate zones like the USA would experience high risks of extreme heat only at and above 3 °C of warming following the global threshold setting applied here. This might be partly due to the choice of the climate hazard indicator used here, which is sensitive to regional differences in temperature variability. As natural variability is higher in temperate regions compared to tropical regions, emergence of 3σ and 5σ will occur later at higher latitudes^33^.

National level risk assessments may differ from such an approach. Specifically, recent heat extremes experienced in western North America^34^, as well as at the Arctic Circle provided a stark reminder of the risks posed by extreme heat in mid and high latitudes. Based on these observed extremes occurring at around 1.2 °C of warming, a regionally informed global assessment of risks can be perceived that assesses “high risks” being reached at 1.5 °C for the USA. Such an assessment is illustrated in Fig. 5f, based on a pessimistic vulnerability assumption and setting the aggregate risk exposure threshold to 5.5% of each country’s 2050 population under SSP2. Risks for other countries and the global level are assessed using the same methodology. The common framework allows to translate such regionally informed threshold selections to other regions and the global level. Such an RFC threshold selection would greatly bring forward the time to risk exceedance everywhere. We find “high risks” for most developing countries as well as the global level already present at 1 °C of warming and global and developing country “very high risks” emerging at 1.5 °C (compare Fig. 5f). Similar arguments could be made based on other risk assessments for India, where potentially deadly heat stress could become commonplace above 1.5 °C^35^.

## Towards increased transparency of climate risks assessments

Communicating the many dimensions of uncertainty while still providing useful information about the risks of future climate change, represents an enduring challenge for scientists^31,32,36^. The objective of the RFC framework, to distil the evolution of complex climate change signals down to a singular colour-coded scale, extends this communication challenge even further. In particular, the transparent integration of uncertainty dimensions affecting relevant risk thresholds—particularly non-climate factors, like socioeconomic scenarios or expert judgements—is key.

However, uncertainty does not always create barriers to the provision of useful, actionable information for decision makers^37^—particularly if that uncertainty is well-understood, and its drivers separated^38^. While this is rarely the case for current applications of risk assessments—commercial users often rely on “black box” approaches^39^—any efforts to identify drivers of uncertainty and highlighting their respective size would significantly improve the wider value of climate risk analytics.

By amending the classification framework to first quantify the localised exposure to the four different RFC risk categories, our analysis has introduced a method to systematically assess how different forms of uncertainty affect the subsequent estimates of aggregate risk associated with extreme future heat. Table 1 presents seven dimensions of uncertainty which have been subsequently identified as relevant, not just for the examination of RFC2, but for all RFCs under the framework employed by the IPCC. The first five uncertainty sources have been explored in Figs. 3–5 and are discussed above, while the issues associated with the spatial patterns of hazard emergence and how the speed of warming affects adaptive capacity are addressed further within Supplementary Information. The rate of change of the climate hazard versus socioeconomic development requires particular consideration, as substantial improvements in adaptive capacity may take well into the second half of the 21st century even under optimistic scenarios of development^22,40^. Risks posed by large near-term increases in climate hazards are therefore substantially higher than if the same hazard level is reached later in the century. Even under the most optimistic scenarios of socioeconomic development globally, and without considering climate-imposed development setbacks, it seems implausible that development can outpace unabated climate change^41^.

**Table 1:** Description of the different forms of uncertainty which contribute towards ambiguity in the final presentation of low, moderate, high and very high risks associated with each of the Reasons for Concern.

Uncertainty source	Explanation	Category	Relative impact on globally aggregated RFCs	Relative impact on global population exposed to extreme heat
(1) Aggregating local risks to national or global risks	What number of people need to exceed the local risk categories, so to achieve exceedance of each of the aggregate risk thresholds?	Expert judgement	Very high	±≈3 billion
(2) Quantifying the importance of vulnerability	How should the gradients of the risk categories vary as a function of vulnerability?	Expert judgement	High	±≈2 billion
(3) Climate model uncertainty	What changes in regional and global temperatures will occur in response to a given greenhouse gas emissions scenario over the 21st century?	Geophysical uncertainty	High	±≈2 billion
(4) Population changes	What changes in the size of regional and global populations will emerge in the 21st century?	Socioeconomic uncertainty	Moderate	±≈500 million
(5) Governance index changes	What changes in global governance will emerge in the 21st century?	Socioeconomic uncertainty	Moderate	±≈500 million
(6) Adaptive capacity	How important is the speed of warming? By how much are local risks reduced, if a hazard threshold is exceeded in 2090, instead of 2050.	Geophysical uncertainty/expert judgement	Low/Moderate	±≈200 million
(7) Spatial patterns of hazard	What differences exist in the relative emergence of local risk thresholds, depending on the type of climate hazard selected?	Geophysical uncertainty/expert judgement	Low	±≈100 million

The final two columns present an estimate of the relative importance of each uncertainty source.

When qualitatively ranking these difference sources of uncertainty on the basis of their relative influence, several clear results emerge. While well-studied factors like climate model uncertainty are of course present, the uncertainties which have fewer quantitative constraints and are often overlooked, dominate. Of particular note, the uncertainty relating to vulnerability—or quantifying what combination of TXx emergence and GI score should be associated with each local risk category—remains not only substantial but also difficult to meaningfully reduce in a globally uniform assessment. However, the final decision of how to aggregate highly heterogeneous regional experiences into a singular global risk classification remains the most subjective, and therefore uncertain, element of the risk assessment. This is illustrated by the very different outcomes resulting from global or nationally-informed threshold settings used in the examples in Fig. 4f, g. Similar examples could be found by accounting for the intergenerational dimensions of exposure to climate risk^42^.

Even though these expert judgements related to the RFC aggregation process are subject to irreducible uncertainties, they can nevertheless be examined both systematically and with transparency (Figs. 4 and 5). Similar principles could equally apply to selecting the RFC categories themselves. For example, if the “extreme weather events” RFC actually considered temperature- and precipitation-related extremes independently, the corresponding risk assessments would likely appear very different, with arguably more useful information being conveyed as a consequence.

The RFC framework communicates scientific understanding about risks relating to varying levels of climate change, and in doing so, represents a critical communication tool for the scientific community^18^. However, any efforts to summarise the multidimensional risk profile of a warming world must confront trade-offs between the simplicity, usefulness and scientific accuracy of this messaging. By presenting a method to systematically evaluate the uncertainties associated with the RFC process, this analysis provides a more rounded understanding of how expert judgements are made, and why subjective choices remain unavoidable when synthesising climate change risks. Our results strengthen claims that “high” risks for extreme heat emerge above 1.5 °C of warming, while showing that exceeding 2 °C would bring the world close to “very high risks” posed by extreme heat^43^. This framework thus substantiates the urgent need for stringent mitigation efforts to limit the risks of extreme heat in the 21st century.

## Methods

### Hazard data

To calculate the hazard, we extract daily temperature data over the period 1861-2100 from the first ensemble member (r1i1p1) of 23 models contributing to the CMIP5 ^44^, with “Historical” and “RCP8.5” simulations from each model concatenated together. The annual maxima of all daily maximum temperatures (hereafter TXx) are then calculated for each location, consistent with recommendations from the Expert Team on Climate Change Detection Indices^45^.

Following previously published methods^6,21,46^, we focus on S/N ratios of change in TXx with future warming, which requires normalising the magnitude of future change against an estimate of internal variability calculated from historical simulations. Using S/N ratios instead of percentages or absolute measures of change is particularly useful to demonstrate when unfamiliar climates and events are realised, a key component of the RFC framework and crucial for adaptation.

Following ref. ^21^, the signal of TXx at each grid cell is calculated as the mean over a running 21-year time window, relative to the mean over the period 1861–1880. The noise term is calculated for each grid cell within each model as the standard deviation over 1901–2000, using “Historical” model simulations detrended with a simple linear fit. Corresponding S/N ratios are calculated for each model at their native resolution first, before being interpolated to a common 2.5° × 2.5° grid thereafter.

The corresponding estimate of global-mean temperature anomalies for each model is also calculated using a 21-year running mean and 1861–1880 baseline. Here, global-mean temperatures refer to the area-weighted average of near-surface air temperatures over all land and ocean regions in each model, with no masking to specific regions with high observational coverage.

Since understanding the risks associated with specific global warming thresholds has been a focus of recent efforts in the climate science community^47–50^, we next identify the 21-year period for which a global warming anomaly of *X* °C is exceeded within each individual model, and remains exceeded until the end of the time-series (2100). We focus on specified half-degree thresholds of warming, *X* = {1.0, 1.5, 2.0, 2.5, 3.0, 3.5}, with corresponding S/N ratios of TXx then extracted for that same 21-year time period.

### Population data

To calculate exposure we obtain spatially explicit population data for the year 2015 from the Center for International Earth Science Information Network database^51^. Preserving the population data at the 0.25° × 0.25° spatial resolution provided, we separate the population of each of 224 separately detectable countries into individual arrays.

We then obtain country-level population projections for each future decade from the SSPs^29^. Because the 2015 population within the SSPs do not actually match observed values, we have to obtain spatially explicit estimates of future population for individual countries in a slightly more complex way: we first calculate the fractional increase in country-level population between 2015 and the future year of interest within the SSP-based model framework, and then multiply each element of the individual gridded arrays of *observed* 2015 population data by this amplification ratio, for each country.

### GI data

As a measure of vulnerability, we use corresponding GI data for each country taken, as is, from Andrijevic et al. See their paper^22^ for further details on the calculations.

We chose to only consider countries which had future country-level projections of both population and the GI under the SSP scenarios. This reduced the population under consideration (in 2015) from 7.2 billion people to 7.03 billion people.

Once this processing was complete, and we had individual arrays of future population for each individual country, we then aggregated the population data up to the coarser 2.5° × 2.5° resolution of the model data, and then found population-weighted estimates of TXx S/N ratios, for each individual country.

## Supplementary information

Supplementary Information

## Data Availability

The CMIP5 model data used to calculate projected global temperature changes in this analysis are available at https://esgf-node.llnl.gov/search/cmip5/. Precomputed extreme temperature indices from the CMIP5 archive are available at https://climate-modelling.canada.ca/climatemodeldata/climdex/. Present-day population data are available at https://sedac.ciesin.columbia.edu/data/collection/gpw-v4/. Future population scenario data are available at https://tntcat.iiasa.ac.at/SspDb. The data to generate the figures used in this analysis can be found at https://git.geo.vuw.ac.nz/harrinlu/NComms2021analysis.

## Code availability

The code to generate the figures used in this analysis can be found at https://git.geo.vuw.ac.nz/harrinlu/NComms2021analysis.
