# Supplementary material for: Quantifying uncertainty in aggregated climate change risk assessments
Source: Nat Commun. 2021 Dec 8;12:7140. doi: 10.1038/s41467-021-27491-2 (PMC8655081; doi:10.1038/s41467-021-27491-2)
Supplement: Supplementary file 1 — Supplementary Information [file 41467_2021_27491_MOESM1_ESM.docx]

**SUPPLEMENTARY INFORMATION for “Quantifying uncertainty in aggregated climate change risk assessments”**

Luke J Harrington^1^, Carl-Friedrich Schleussner^2,3^ & Friederike E L Otto^4^

^1^New Zealand Climate Change Research Institute, Victoria University of Wellington, Wellington 6012, New Zealand.

^2^Climate Analytics, 10969 Berlin, Germany

^3^IRI THESys, Humboldt University, Berlin, Germany

^4^Grantham Institute, Imperial College London, UK

---

***Differences between heat-related risks in 2050 versus 2090***

Two key differences emerge, when the results presented in the main article are instead examined for the year 2090: (1) the changes in both population size and quality of governance within the SSP scenarios; and (2) changes to the local risk gradients - these have been amended to reflect the fact that the risks associated with a 2°C warming in global temperatures will be less severe if communities have another four decade to prepare for such changes.

Specifically, we modify the default profile of risk (for a 2°C world under SSP2, but for the year 2090), as demonstrated in Supplementary figure 1. The upwards shifts of the thresholds separating the moderate/high, and high/very high risk categories, are intended to reflect a greater capacity to cope with extreme heat, if exposure to a given threshold of global warming emerges gradually over the entire twenty-first century, rather than emerging rapidly by 2050. We reiterate that this estimated change in vulnerability owing to a greater adaptive capacity is also very uncertain, which remains captured by the alternative variants presented in the bottom row of Supplementary figure 2.

When comparing Supplementary figure 1 with Figure 2 of the main article, we find the adjustments associated with moving to 2090 data under SSP2 include a broad shift towards countries exhibiting better levels of governance. This, coupled with the changes associated with improved adaptive capacity, lead to a ten-fold reduction in the number of people within the ‘very high’ risk category, as well as a billion fewer people within the ‘high’ risk category. Similar changes are also found when exploring the full uncertainty range in Supplementary figure 2.


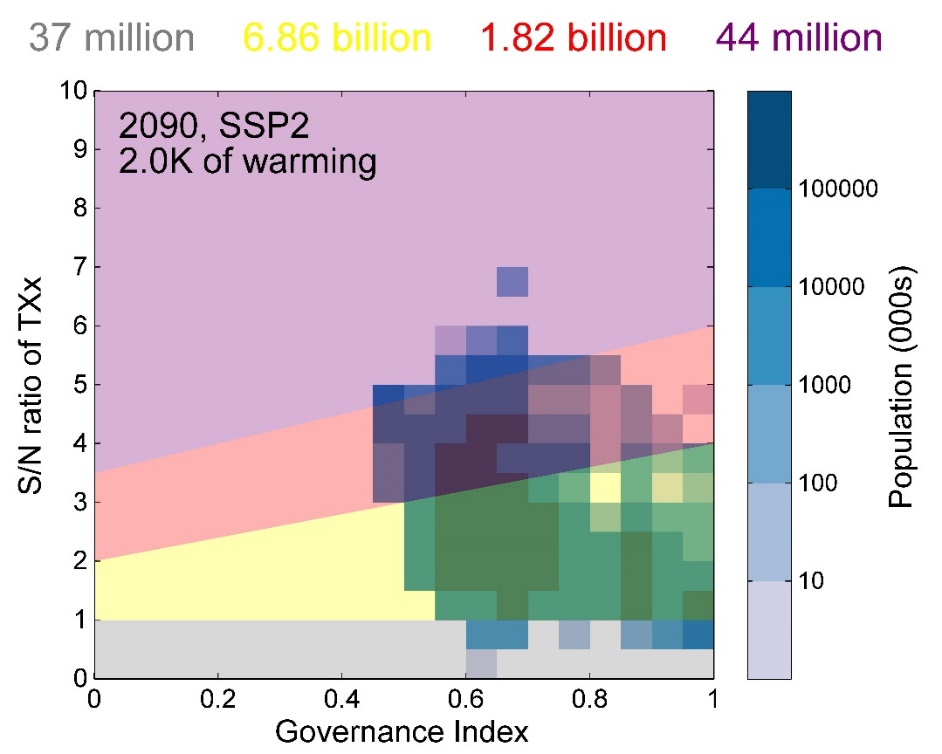


**Supplementary Figure 1:** Same as Figure 2 of the main article, but using 2090 data from SSP2, alongside an amended background risk profile, which reflects the adaptive capacity gained by experiencing a given threshold of warming at the end of the 21^st^ century, rather than in 2050.

**CMIP5 MODEL UNCERTAINTY**


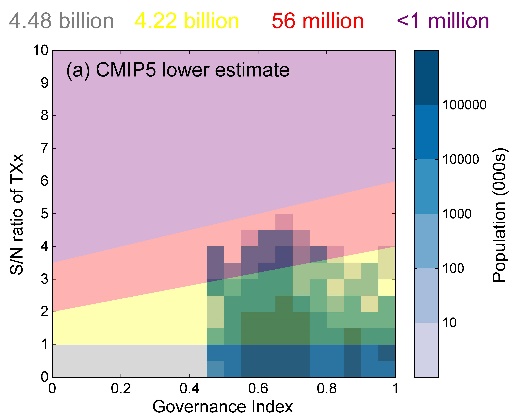

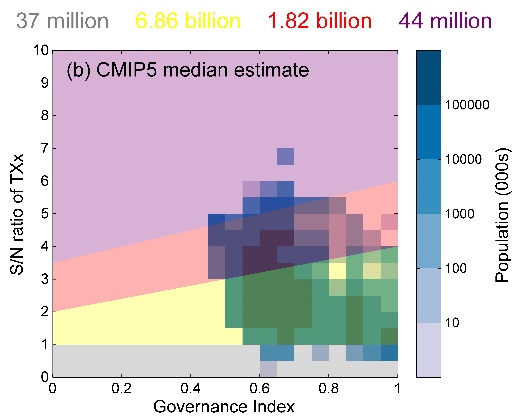

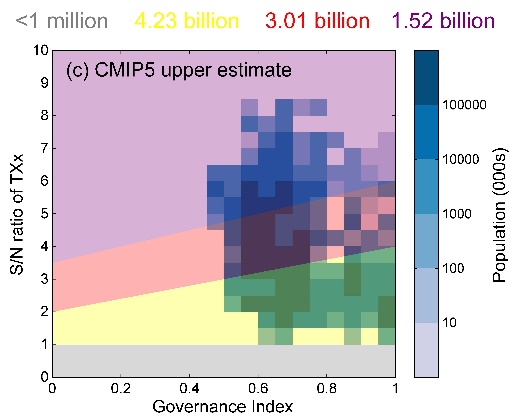


**EMISSIONS SCENARIO UNCERTAINTY**


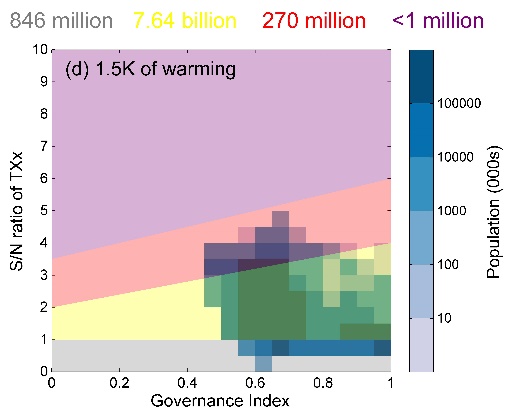

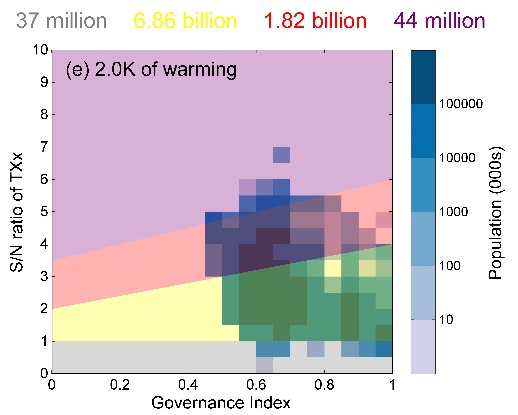

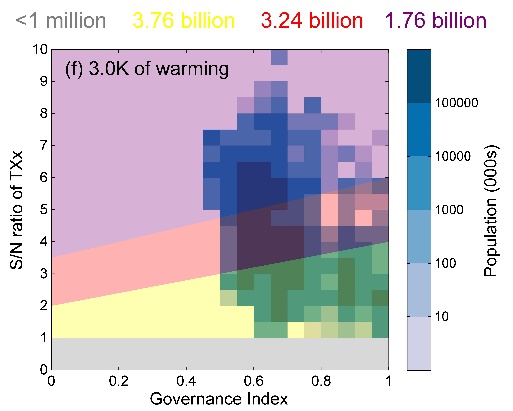


**SOCIO-ECONOMIC SCENARIO UNCERTAINTY**


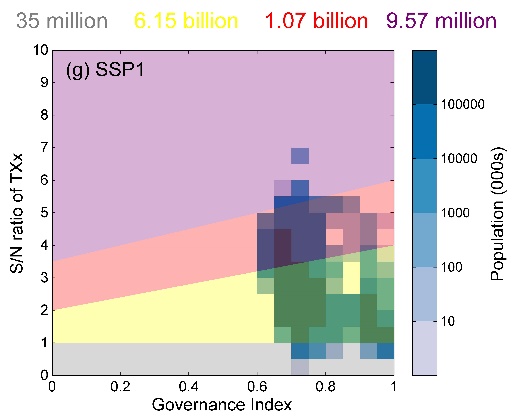

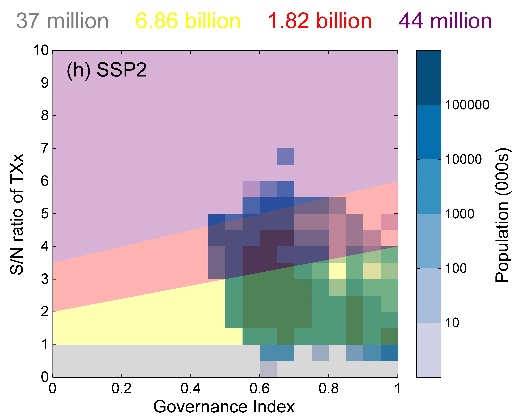

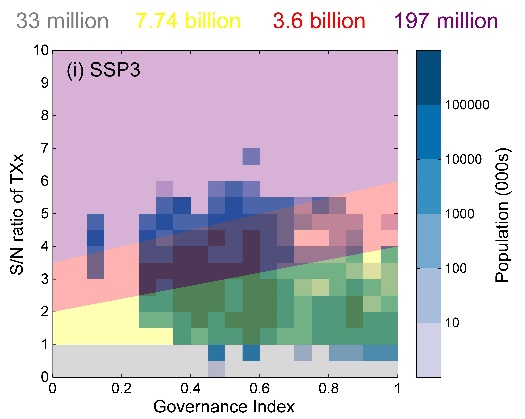


**VULNERABILITY UNCERTAINTY**


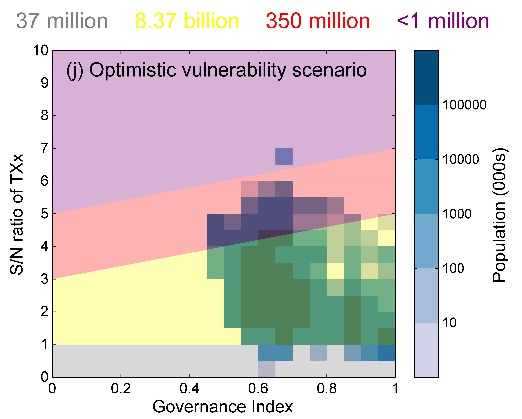

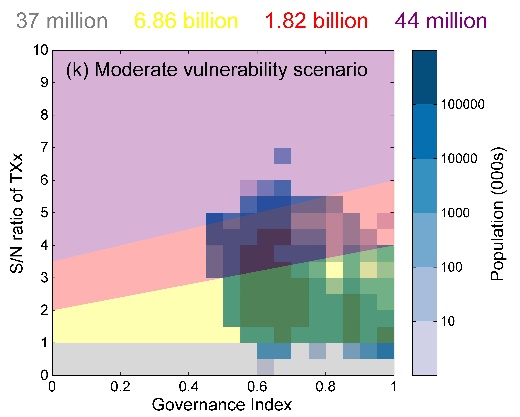

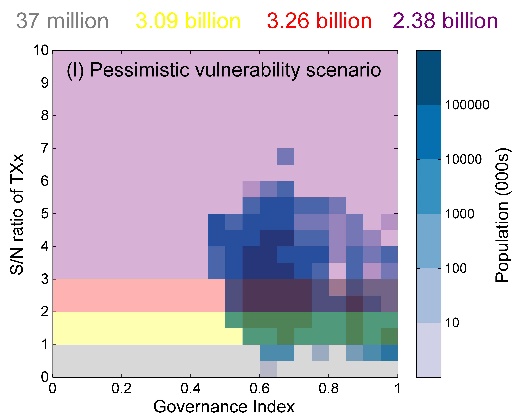


**Supplementary Figure 2:** Same as Figure 4 of the main article, but using 2090 as the year to extract the population and GI data from the relevant SSP scenarios. Also noted is the default profile of risk (middle columns) use an amended thresholds for the local risk categories, to reflect the increased adaptive capacity by 2090.

***Uncertainty associated with the spatial patterns of the emergent hazard***

We recognise that some metrics of the risks associated with extreme heat focus only on the signal of change, rather than contextualising that signal of change relative to past experiences (signal-to-noise ratio). Furthermore, we acknowledge that we have chosen patterns of signal-to-noise ratios in TXx as being representative of the spatial patterns of *all emergent hazards* associated with extreme weather events. While the reasons for only choosing this single variable as a proxy are discussed in depth within the main article, we nevertheless want to explore what differences might emerge if other spatial patterns of hazard emergence are considered.

To do this, we hereafter repeat the analysis of the main article, but using only the signal of TXx changes with warming (Supplementary figure 3a) rather than the signal-to-noise ratios (Supplementary figure 3b) used for the main analysis. These two patterns of change are as different as might be conceivably expected, while still reflecting plausible geographic patterns of future hazard changes associated with “extreme weather events”. Therefore, any differences in the results presented here can be considered the plausible range of uncertainty in the spatial patterns of hazard emergence, relative to the S/N-based results of the main article.

Supplementary figure 4 presents the same nine panels as Figure 4a-i within the main article, considering the CMIP5 uncertainty bounds (panels a-c), emissions scenario uncertainty (panels d-f) and socio-economic scenario uncertainty (panels g-i), relative to a 2°C world in 2050 under SSP2, *but looking at the absolute change in TXx as the relevant hazard on the y-axis.* Because of this shift from a normalised hazard metric to an absolute hazard metric, we cannot simply map the risk thresholds accordingly: a +1σ or +2σ change in TXx is not equivalent to a +1°C or +2°C increase in TXx, for example. So we emphasise that the panels in Supplementary figure 4 should only be compared with Figure 4 of the main article, with respect to how the clusters of GI/TXx data vary across the nine panels. When viewed under this lens, we see that the majority of changes found when exploring the dimensions of uncertainty using S/N ratios of TXx are very similar to those patterns which emerge when looking at absolute signals of TXx instead. This is unsurprising, by the very nature of aggregating the exposure of the entire population into a single graphic.


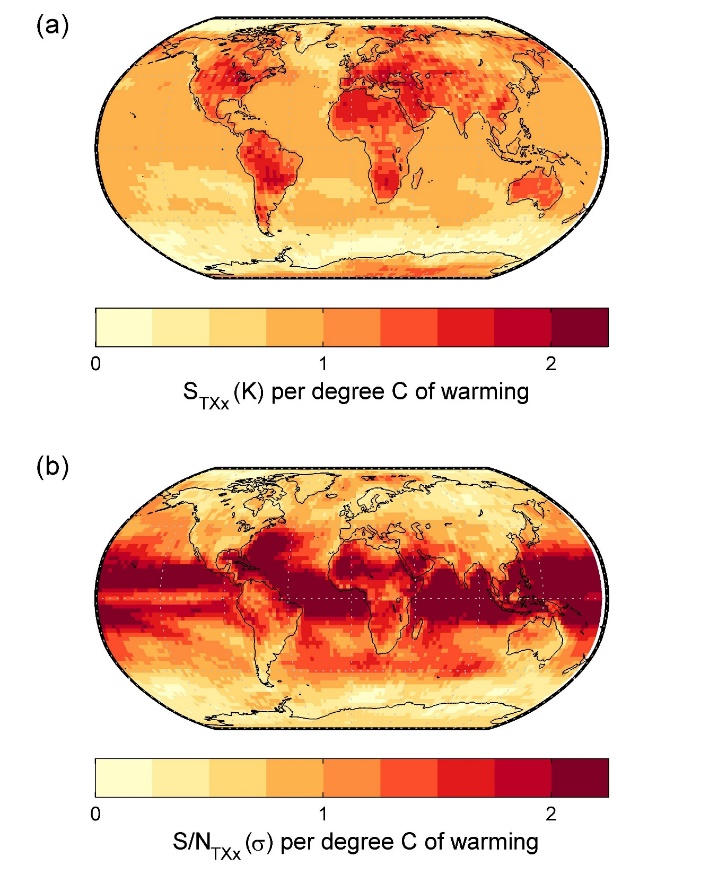


**Supplementary Figure 3: (a)** Multi-model medial spatial patterns of the change in TXx per degree C of warming under future warming scenarios. **(b)** Same as panel (a), but showing the spatial patterns of signal-to-noise ratios of TXx: these patterns are used for the bulk of the analysis presented within the main article.

**CMIP5 MODEL UNCERTAINTY**


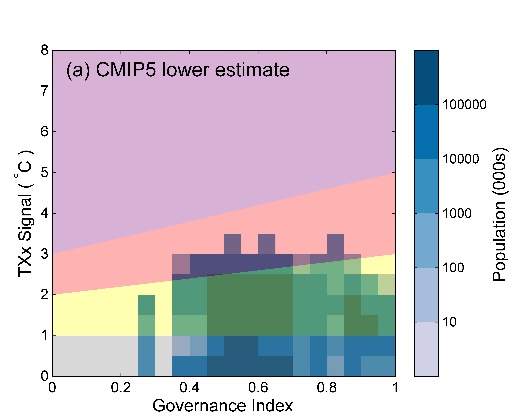

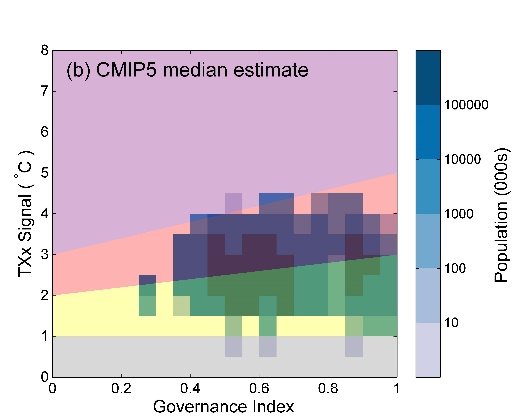

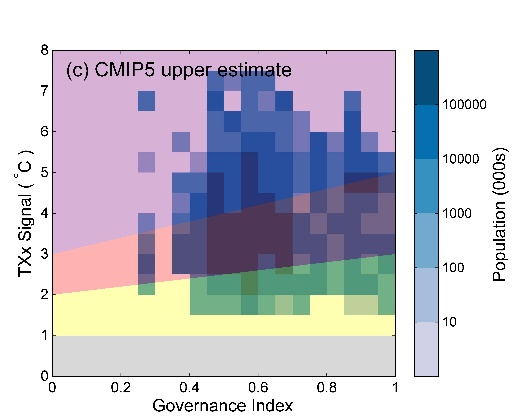


**EMISSIONS SCENARIO UNCERTAINTY**


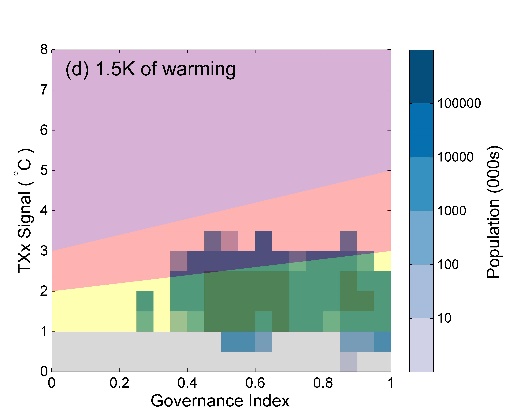

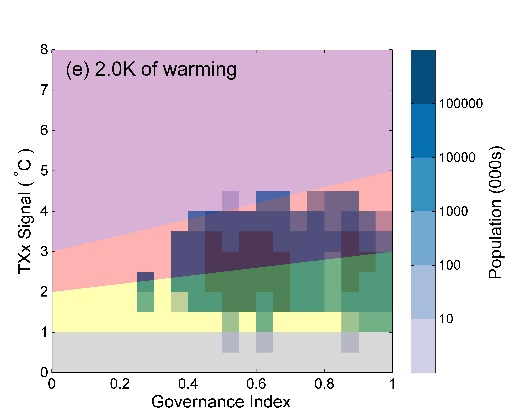

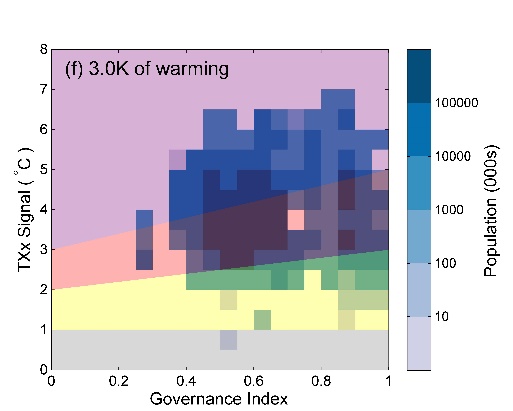


**SOCIO-ECONOMIC SCENARIO UNCERTAINTY**


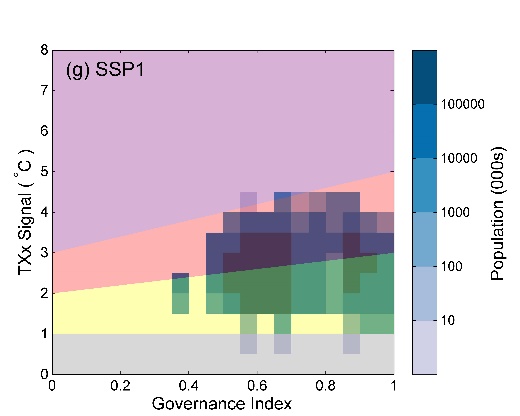

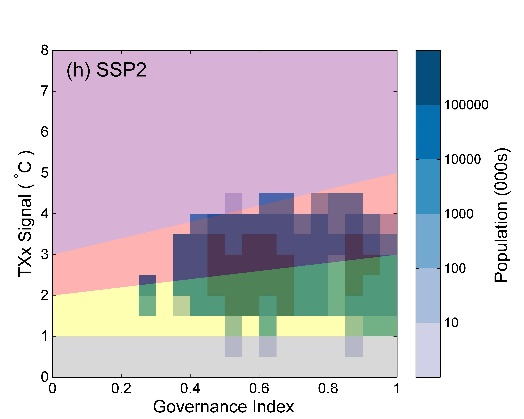

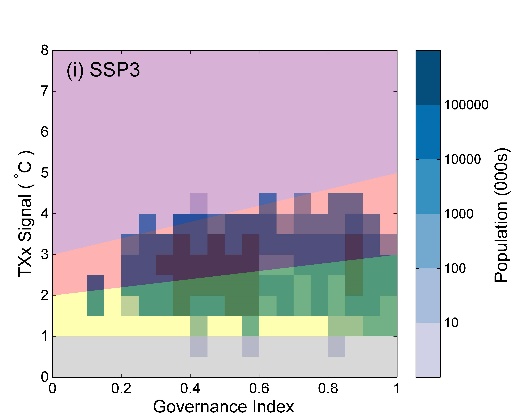


**Supplementary Figure 4:** Same as Figure 4 of the main article, but using the *signal* of TXx to represent the emergent climate hazard (y-axis) instead. We further note that the background risk panels are not actually used to calculated aggregate risks in this case – this is because translating these risk thresholds to a new hazard, when the new hazard changes from a normalised to an absolute metric, is unclear.

***Aggregated risk estimates at the country-scale***

Supplementary figures 5-10 respectively show, for each of the six individual countries considered in figures 5f-g – USA, India, Indonesia, Democratic Republic of Congo, Nigeria & Brazil - the number of people experiencing local risks equal to, or worse than, “moderate”, “high” or “very high” categories, at different thresholds of global temperature rise, and considering all dimensions of uncertainty explored in figures 4, and Supplementary figures 1-4. As with figures 5a-c of the main article, in each figure, darker markers show the outcomes for the median CMIP5 results, and using a “moderate” vulnerability assumption; the lighter colours show the range of outcomes associated with the 10^th^ percentile and 90^th^ percentile of CMIP5 model output.

As with figures 5a-c of the main article, country-level exposure thresholds have also been chosen to quantify aggregate risk categories. Here, the dashed, straight and dotted horizontal grey lines respectively denote the ‘risk-averse’, ‘risk-neutral’ and ‘risk-tolerant’ exceedance thresholds, which have been chosen to align with 7.5%, 15% and 30% of each country’s population at 2050 under a ‘middle-of-the-road’ SSP2 scenario.


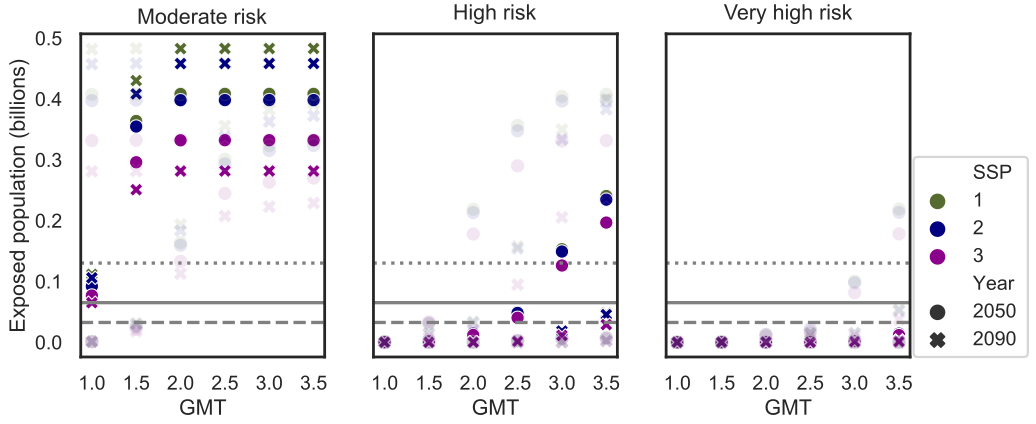


**Supplementary Figure 5:** Same as Figures 5a-c of the main article, but showing outcomes for the USA.


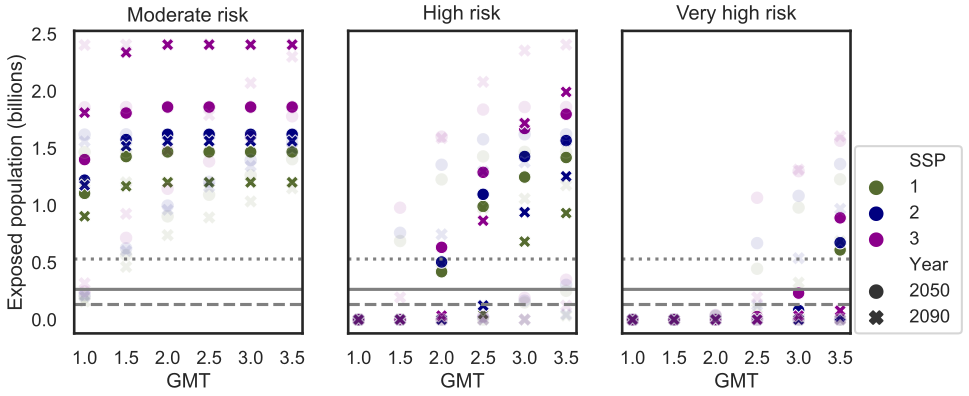


**Supplementary Figure 6:** Same as Figures 5a-c of the main article, but showing outcomes for India.


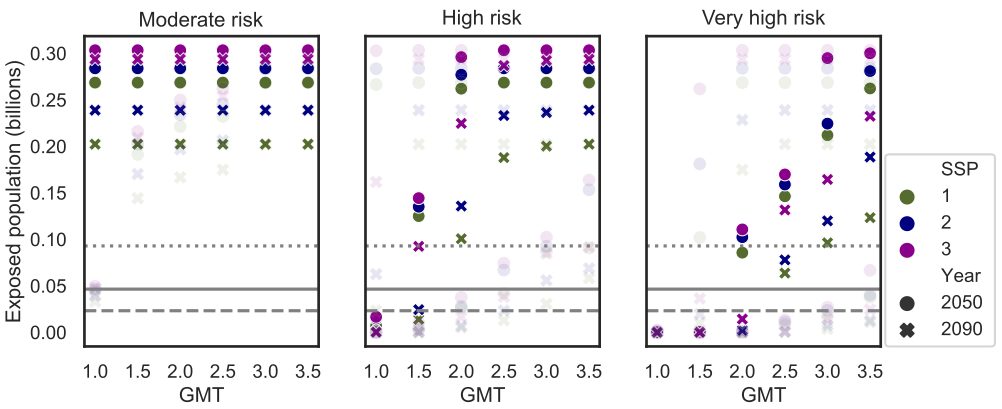


**Supplementary Figure 7:** Same as Figures 5a-c of the main article, but showing outcomes for Indonesia.


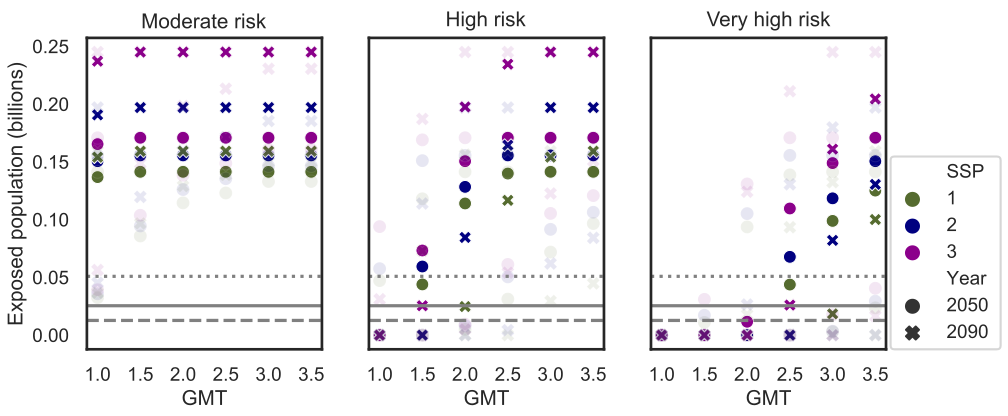


**Supplementary Figure 8:** Same as Figures 5a-c of the main article, but showing outcomes for the Democratic Republic of Congo (DRC).


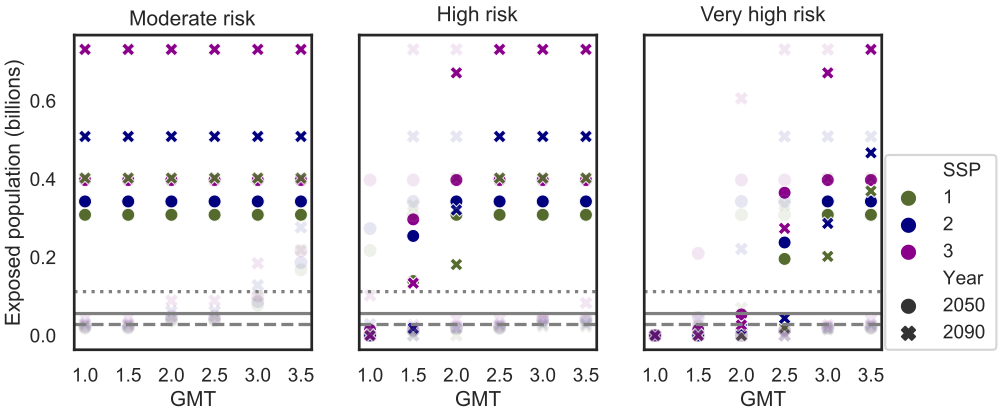


**Supplementary Figure 9:** Same as Figures 5a-c of the main article, but showing outcomes for Nigeria.


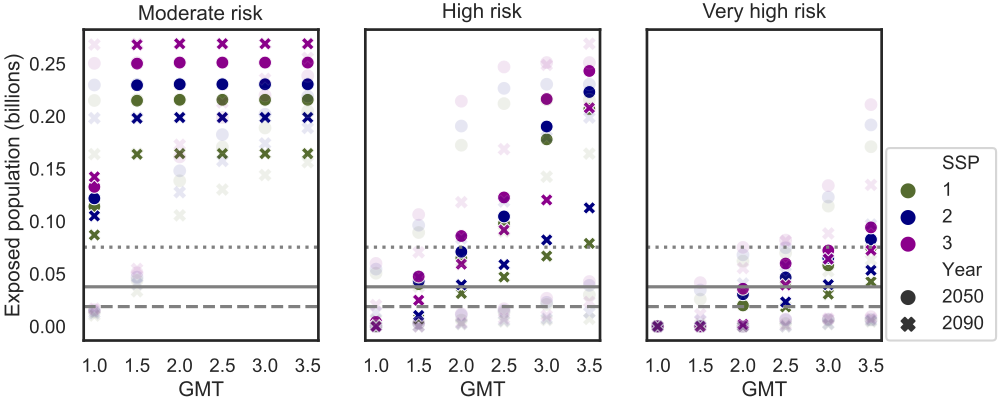


**Supplementary Figure 10:** Same as Figures 5a-c of the main article, but showing outcomes for Brazil.
